# A model for shared clinical care in the COVID-19 crisis

**DOI:** 10.1017/ice.2020.363

**Published:** 2020-07-23

**Authors:** Katrin Gillis, Peter Van Bogaert, Hilde Servotte, Serge Lievens, Henk Cuvelier, Philip Nieberding, Veroniek Saegeman

**Affiliations:** 1 Department of Health Care, Odisee University College, Sint-Niklaas, Belgium; 2 Antwerp University, Centre for Research and Innovation in Care, Antwerp, Belgium; 3 Vzw Samen-Ouder, Sint-Niklaas, Belgium; 4 AZ Nikolaas, Department of Nursing, Sint-Niklaas, Belgium; 5 AZ Nikolaas, Medical Department, Sint-Niklaas, Belgium; 6 AZ Nikolaas, Department of Microbiology and Infection Control, Sint-Niklaas, Belgium

*To the Editor—*During the coronavirus disease 2019 (COVID-19) pandemic, it has become clear that the morbidity and mortality from COVID-19 are higher among elderly patients than in younger age groups in most Western countries.^1,2^ Moreover, elderly patients often live together in residential facilities, which increases their infection risk. In Belgium, very soon after the onset of the pandemic, a ban was put on family visits to residents of these facilities; this was one of the first measures before the start of a general lockdown. In Flanders, the Dutch-speaking part of Belgium, the government decided to have all residents and employees tested. Federal recommendations mentioned quarantine of severe acute respiratory coronavirus virus 2 (SARS-CoV-2)–positive residents on designated cohort units within residential care facilities.^3^

**Fig. 1.**
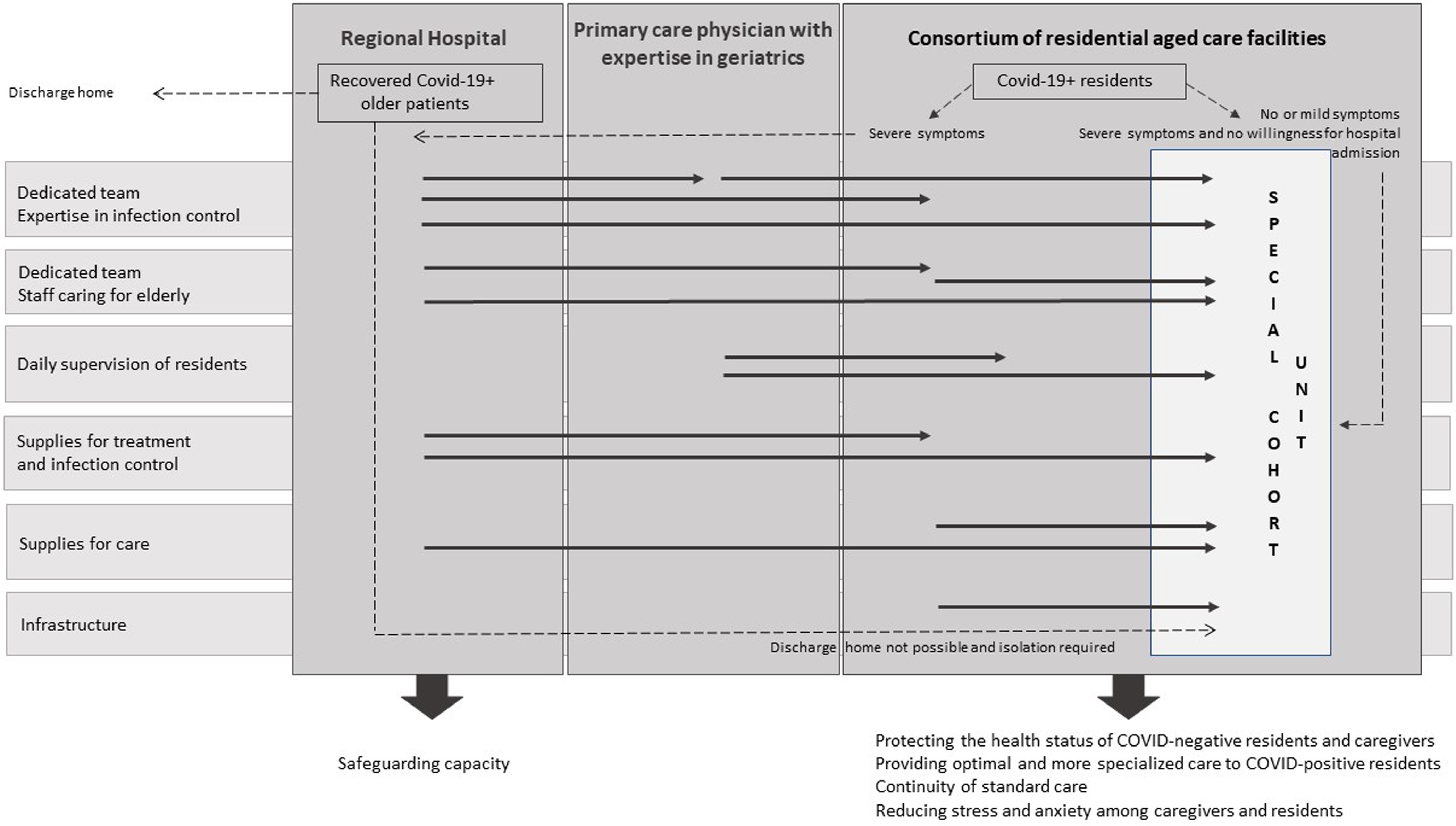
Model for shared clinical care in the management of SARS-CoV2.

However, the Flemish Agency for Care approved the initiative of a group of residential care facilities to install a centralized special cohort unit for older adults with COVID-19. SARS-CoV-2–infected residents from multiple residential facilities were transferred to this unit, as well as SARS-CoV-2–infected elderly adults who were discharged from hospitals but were too frail to return home. This special cohort unit was located in the Waasland region, and it provided shelter for residents from a group of 6 residential facilities that are part of a consortium (580 residents). A close collaboration was set up with an 810-bed regional hospital and coordinating physician of the primary care zone. The unit provided care for 10 residents, with the possibility to scale up with 10 additional places on standby. Four essential conditions had to be fulfilled before starting up such a unit: (1) the availability of infrastructure to enable barrier nursing; (2) the availability of a dedicated team with sufficient expertise in infection control and care for older adults; (3) the availability of sufficient supplies for care, treatment, and infection control; and (4) the possibility of fulfilling conditions 1–3 without influencing the regular residential care of the other units or the hospital. Due to the unprecedented and unseen impact of this crisis, the regional partners decided to apply the format of shared clinical care and provided the necessary infrastructure, materials, knowledge, and staffing (Fig. 1).

One of the residential facilities had an empty unit available with closed corridors (due to upcoming renovations). The unit had a separate entrance only accessible from the outside of the building that could be used by caregivers employed on the designated unit. Next, the needed protective and medical equipment (eg, IV therapy) were supplied by the regional hospital. Equipment for nursing care, maintenance, and meal distribution were provided by the other residential facilities of the consortium. Disposable materials were used as much as possible to limit the risk of iatrogenic infections, such as use of no-rinse disposable wash gloves during morning care and of disposable cutlery for meals.

The nursing team of the cohort unit was composed of 8 volunteer nurses and 2 nurse assistants (ie, 9 full-time equivalents, FTE). To ensure continuity on the other regular units in the residential facilities, vacancies created due to the absence of permanent staff were filled by nurses from the hospital. Seven of the nurses had bachelor’s degrees, with an average work experience of 22 years, a level of experience and education above the average in regular units in Flemish residential facilities.^4^ The voluntary character and the level of education enabled nurses to work autonomously in this cohort unit during this crisis. Still, nurses had little experience with the principles of barrier nursing on cohort units, and they were therefore supported by hospital staff. In addition, the hospital also outsourced an infection prevention physician to share insights on infection prevention. Advice and procedures regarding personnel protection for healthcare workers were shared. Furthermore, a primary-care physician with specific expertise in infections in older adults was designated to the ward for the daily supervision of the medical follow-up of the residents on the unit.

Over 52 days, 15 residents were admitted to the special cohort unit. Among them, 13 were residents of the consortium and 2 individuals were admitted because they could not return home after discharge from the hospital. Unfortunately, 6 of these patients died. After 30 days, all members of the nursing team tested negative for COVID-19.

Due to the format of shared clinical care between the regional hospital, the consortium and the primary care zone, the conditions assumed to make such unit possible were achieved. The special cohort unit had sufficient protective and medical equipment; specialized expertise was available; barrier nursing was successfully employed; and routine activity on the other units was assured. This far-reaching collaboration made it possible to remove SARS-CoV-2–positive residents from residential facilities early, therefore avoiding further spread and preserving and safeguarding the capacity of the regional hospital. During the COVID-19 pandemic, the developed and implemented shared primary and secondary clinical care model has proven to be a sound solution for keeping the situation in our region under control. The model is adaptable to local contexts and may be an inspiration for other regions in different countries.
